# Loss of *Atg2b* and *Gskip* Impairs the Maintenance of the Hematopoietic Stem Cell Pool Size

**DOI:** 10.1128/MCB.00024-21

**Published:** 2022-01-20

**Authors:** Shun-suke Sakai, Atsushi Hasegawa, Ryosuke Ishimura, Naoki Tamura, Shun Kageyama, Satoko Komatsu-Hirota, Manabu Abe, Yiwei Ling, Shujiro Okuda, Manabu Funayama, Mika Kikkawa, Yoshiki Miura, Kenji Sakimura, Ichiei Narita, Satoshi Waguri, Ritsuko Shimizu, Masaaki Komatsu

**Affiliations:** a Division of Clinical Nephrology and Rheumatology Kidney Research Center, Niigata University Graduate School of Medical and Dental Sciences, Niigata, Japan; b Department of Molecular Hematology, Tohoku University School of Medicine, Sendai, Japan; c Department of Physiology, Juntendo University Graduate School of Medicine, Tokyo, Japan; d Department of Anatomy and Histology, Fukushima Medical University School of Medicine, Hikarigaoka, Fukushima, Japan; e Department of Animal Model Development, Brain Research Institute, Niigata University, Niigata, Japan; f Medical AI Center, Niigata University School of Medicine, Niigata, Japan; g Division of Bioinformatics, Niigata University Graduate School of Medical and Dental Sciences, Niigata, Japan; h Research Institute for Diseases of Old Age, Juntendo University Graduate School of Medicine, Tokyo, Japan; i Laboratory of Proteomics and Biomolecular Science, Biomedical Research Core Facilities, Juntendo University Graduate School of Medicine, Tokyo, Japan

**Keywords:** hematopoietic stem cell, hematopoiesis, ATG2B, GSKIP, autophagy, myeloproliferative neoplasm

## Abstract

A germ line copy number duplication of chromosome 14q32, which contains *ATG2B* and *GSKIP*, was identified in families with myeloproliferative neoplasm (MPN). Here, we show that mice lacking both *Atg2b* and *Gskip*, but not either alone, exhibited decreased hematopoiesis, resulting in death *in utero* accompanied by anemia. In marked contrast to MPN patients with duplication of *ATG2B* and *GSKIP*, the number of hematopoietic stem cells (HSCs), in particular long-term HSCs, in double-knockout fetal livers was significantly decreased due to increased cell death. Although the remaining HSCs still had the ability to differentiate into hematopoietic progenitor cells, the differentiation efficiency was quite low. Remarkably, mice with knockout of *Atg2b* or *Gskip* alone did not show any hematopoietic abnormality. Mechanistically, while loss of both genes had no effect on autophagy, it increased the expression of genes encoding enzymes involved in oxidative phosphorylation. Taken together, our results indicate that *Atg2b* and *Gskip* play a synergistic effect in maintaining the pool size of HSCs.

## INTRODUCTION

Myeloproliferative neoplasms (MPNs) belong to a related group of blood cancers characterized by clonal expansion of hematopoietic stem cell (HSC)-derived cells in one or more hematopoietic lineages (1, 2). The key clinicopathological entities of MPNs are chronic myeloid leukemia, polycythemia vera, essential thrombocythemia, and primary myelofibrosis, all of which are usually caused by specific somatic mutations (1, 2). It is proposed that by disturbing various cellular mechanisms, including signaling pathways, autophagy, and cellular metabolism, MPN-initiating mutations transform HSCs into MPN stem cells that possess a survival advantage over normal HSCs (3–10). In many cases, patients with early-stage disease are clinically unremarkable and remain undiagnosed for months or years, but during this time the abnormal cells gradually replace normal blood cells and occasionally transform into malignant cells resembling genuine acute leukemic cells (1, 2).

*ATG2B* and *GSKIP* are located on chromosome 14q32, where a germ line tandem duplication of a 700-kb region was identified in the pedigrees of four large West Indian families with MPNs (11). Genetic analyses of the families revealed that germ line duplication of *ATG2B* and *GSKIP* conferred a risk of familial myeloid malignancies, and overexpression of *ATG2B* and *GSKIP* in HSCs enhanced hematopoietic progenitor differentiation (11). Both genes were also highly expressed in *de novo* acute myeloid leukemias (AMLs) (12). Meanwhile, a genetic analysis of the pedigree of a North American family with MPNs recently showed that germ line duplication of *ATG2B* and *GSKIP* genes was not required for the development of familiar myeloid malignancy syndromes associated with duplication of chromosome 14q32 (13). The involvement of *ATG2B* and *GSKIP* in hematopoiesis and the development of MPNs and/or AMLs is thus controversial.

The protein encoding *ATG2B* is a core ATG protein that participates in autophagy (14). Autophagy is a system in which an isolation membrane/phagophore that forms in the vicinity of the endoplasmic reticulum (ER) is elongated and a portion of its cytoplasm is sequestered into autophagosomes, which are then transported to lysosomes for degradation (15). The proteins involved in the formation of autophagosomes are called core ATG proteins, each of which consists of six functional units. When the ULK1 protein kinase complex1 is translocated to the ER subdomain, PI3K complex I is recruited and PI(3)P production increases. ATG18 homologues WIPI1 to WIPI4 bind to PI(3)P and accumulate with their binding partners, ATG2A and ATG2B. ATG2A and ATG2B may have redundant autophagy-related functions in mammals (16), and both connect the ER to the isolation membrane/phagophore and transport lipids (16, 17). ATG9A, a membrane protein, transiently accumulates in the isolation membrane/phagophore and scrambles phospholipids transported by ATG2 from the ER to the cytoplasmic layer of the isolation membrane/phagophore (18, 19). ATG12 and ATG5 are covalently bound to each other via a ubiquitin-like conjugation reaction (20, 21). The ATG12-ATG5 conjugate forms a complex with ATG16L and localizes to the isolation membrane/phagophore, which determines the location of amide bond formation between LC3 family proteins and phosphatidylethanolamine (PE). An LC3-PE protein called LC3-II localizes on the isolation membrane/phagophore and promotes autophagosome formation through membrane perturbation (22).

*Gskip* encodes glycogen synthase kinase 3β (GSK3β) interaction protein (GSKIP). This protein is an A-kinase anchoring protein for GSK3β and protein kinase A (PKA) that regulates or facilitates their kinase activity toward their targets (23–25). GSKIP directly interacts with GSK3β, a component of the canonical Wnt signaling pathway, and plays a critical role in embryonic development and functions as a negative regulator of GSK3β (26). *Gskip*-deficient mouse embryos showed incomplete closure of the palatal shelves accompanied by delayed ossification along the fusion area of the secondary palatal bones, probably due to modulation of GSK3β, causing lethality at birth (27).

Copy number variation of chromosome 14q32 upregulates the gene expression of *WIPI1*, an *ATG18* homologue, and is associated with increased conversion of LC3-I to LC3-II, suggesting that autophagic activity is increased in patients with MPNs (11). ATG2B is directly involved in autophagy, and GSK3β also regulates autophagy through AMPK activation and/or mTORC1 inactivation (28). However, no studies have demonstrated a biological association between ATG2B and GSKIP. Here, we investigated how simultaneous loss of the *Atg2b* and *Gskip* genes affected maintenance of the pool size of HSCs in mice, as well as autophagy and gene expression in *Atg2B Gskip*-deficient human leukemia cell lines.

## RESULTS

### Generation of *Atg2b Gskip* double-knockout mice.

**FIG 1:**
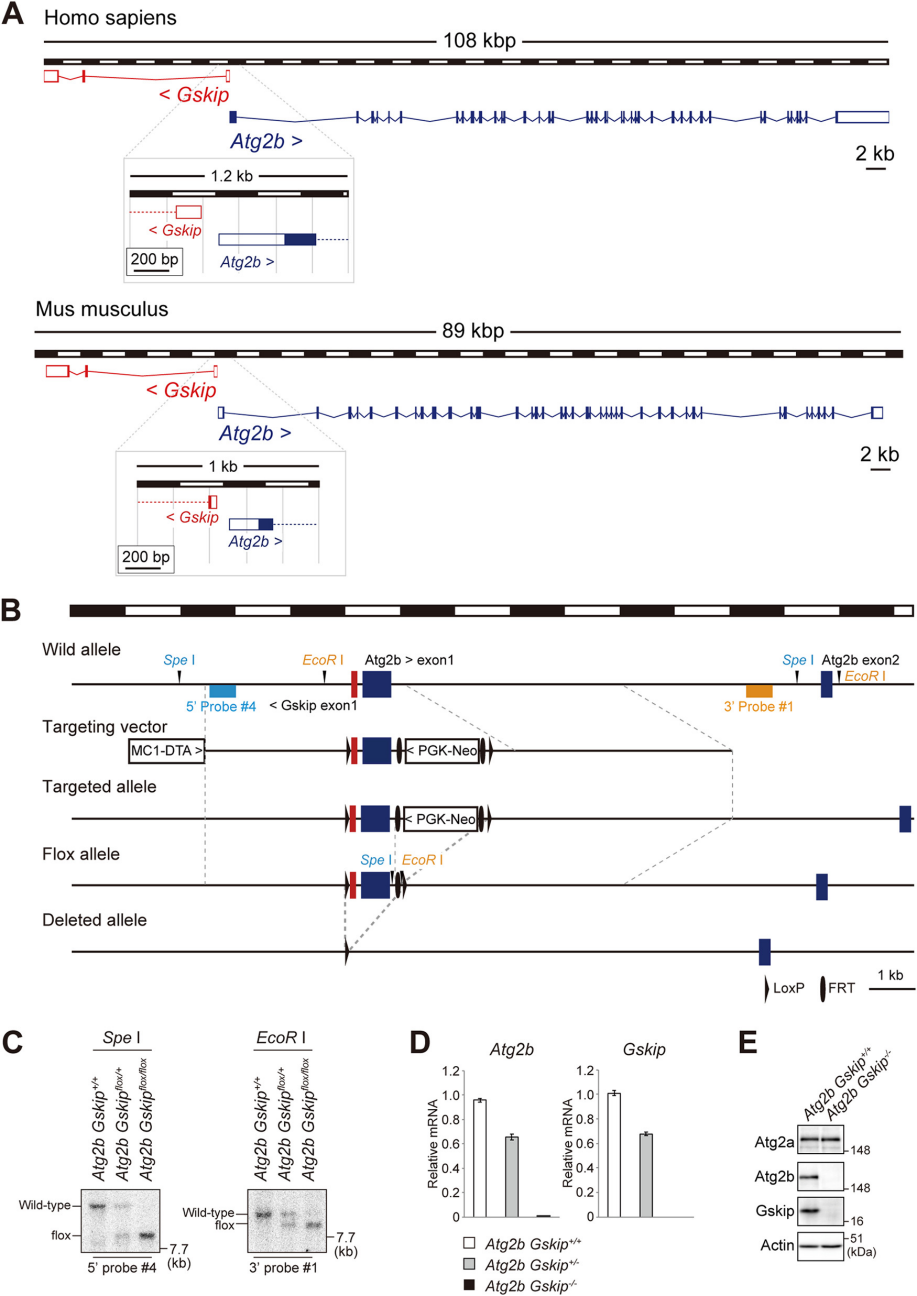
Generation of *Atg2b Gskip^−/−^* mice. (A) Genomic structures of human and mouse *ATG2B* and *GSKIP*. (B) Schematic representation of the targeting vector and the targeted allele of the *Atg2b* and *Gskip* genes. SpeI, SpeI site; EcoRI, EcoRI site; Neo, neomycin resistance gene cassette; DTA, diphtheria toxin gene. (C) Southern blot of genomic DNAs extracted from mouse tails. Wild-type and flox alleles are detected as 16.5- and 9-kb bands or 10.5- and 8.9-kb bands, respectively. Expression of *Atg2b* and *Gskip* transcripts and proteins in double-deficient mouse embryonic fibroblasts. (D) Real-time PCR analysis. Total RNA was prepared from indicated genotype mouse embryonic fibroblasts (MEFs). Values were normalized against the amount of mRNA in the wild-type MEFs. (E) Immunoblot analysis. Lysates prepared from indicated genotype MEFs were subjected to SDS-PAGE, followed by immunoblot analysis with the indicated antibodies. Data shown are representative of three separate experiments.

**FIG 2:**
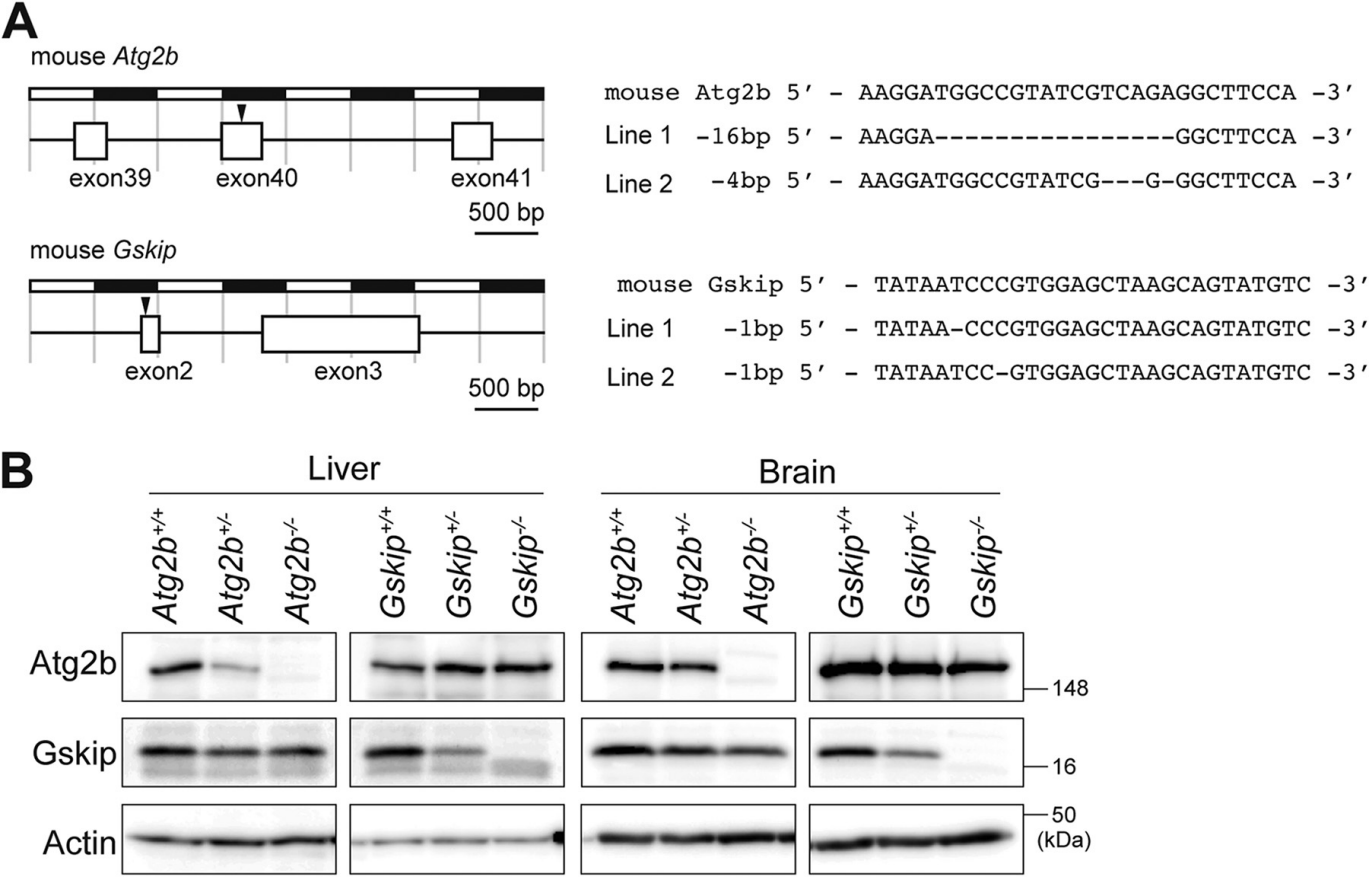
Generation of *Atg2b* and *Gskip* single-knockout mice. (A) Positions of guide RNA (gRNA)-targeted sequences in mouse *Atg2b* and *Gskip* and sequences of the mutant alleles from *Atg2b*- and *Gskip*-deficient mice. Each mutation is highlighted. (B) Immunoblot analysis. Homogenates of the livers and brains of mice with the indicated genotypes were subjected to SDS-PAGE, followed by immunoblot analysis with anti-Atg2b, anti-Gskip, and antiactin antibodies.

As in the human genome, mouse *Atg2b* is located next to *Gskip* on chromosome 12, and both exon1 regions are adjacent (Fig. 1A). To investigate the function of Atg2b and Gskip in mice, a targeting vector was constructed by insertion of *loxP* sequences prior to and after exon1 of the *Atg2b* gene (prior to and inside exon1 of the *Gskip* gene) (Fig. 1B). Germline transmission was confirmed by Southern blotting (Fig. 1C). To delete the neomycin resistance gene, mice with mutant alleles were bred with *PGK-FLPo* transgenic mice (MGI identifier [ID] 4415609). The transgenic mice expressed the mouse codon-optimized FLP recombinase under the direction of the mouse phosphoglycerate kinase 1 promoter. When crossed with a strain containing an FLP recombination target (FRT) site-flanked sequence, FLPo recombinase activity was detected in all cells, with complete recombinase-mediated excision of the target. The resulting progeny containing the *Atg2b Gskip^flox^* allele were bred with *EIIa*-Cre transgenic mice (MGI ID 2137691) to obtain *Atg2b Gskip^+/−^* heterozygous mice. *EIIa*-Cre mice carry a *cre* transgene under the control of the adenovirus *EIIa* promoter, which targets expression of Cre recombinase to the early mouse embryo. These mice are useful for germ line deletion of *loxP*-flanked genes. The heterozygotes were crossbred with each other to generate *Atg2b Gskip* double-knockout mice. We confirmed loss of *Atg2b* and *Gskip* transcripts, as well as that of both proteins, in *Atg2b Gskip* double-deficient mouse embryonic fibroblasts (MEFs) (Fig. 1D and E). As controls for *Atg2b Gskip^−/−^* mice, we also developed *Atg2b* and *Gskip* single-knockout mice by Cas9-CRISPR technology. We targeted exon40 of *Atg2b* and exon2 of *Gskip* so as not to affect the expression of either gene (Fig. 2A). The generated *Atg2b*-deficient mice had 16-bp (line 1) and 4-bp (line 2) deletions of exon40, and the *Gskip*-deficient mice had a 1-bp deletion of exon2 (line1, c.33delT; line 2, c.36delC) (Fig. 2A). We used line 2 as *Atg2b* knockout mice and line 1 as *Gskip* knockout mice, and we verified normal levels of the Atg2b and Gskip proteins in the *Gskip* and *Atg2b* knockout mice, respectively (Fig. 2B).

### Morphological analysis of *Atg2b Gskip* double-knockout mice.

**FIG 3:**
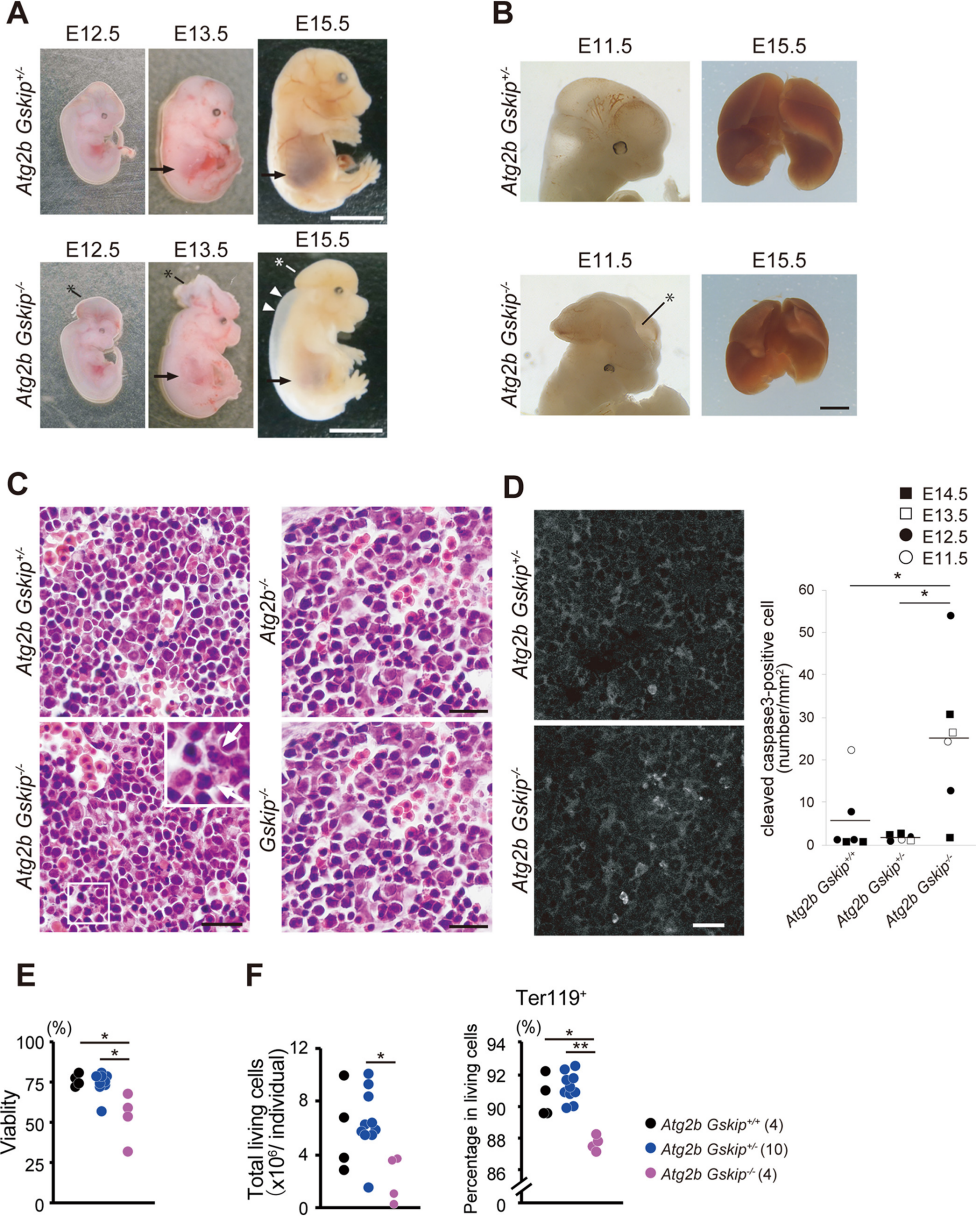
Developmental defects of *Atg2b Gskip^−/−^* mice. (A and B) Representative photographs of *Atg2b Gskip* double-knockout embryos and heterozygous embryos at E12.5, E13.5, and E15.5. *Atg2b Gskip^−/−^* embryos exhibited exencephaly (asterisks in panels A and B) and edema (arrowheads). An anemic phenotype can be seen throughout the body, appearing at E15.5, and in liver areas (arrow). A representative liver of an *Atg2b Gskip^−/−^* embryo is smaller than the liver of a heterozygous embryo (B). Bars, 5 mm (A) and 1 mm (B). (C) HE staining in fetal liver sections of indicated genotypes. Bar, 20 μm. (D) Immunohistofluorescence with anti-cleaved caspase-3 antibody in fetal liver sections from *Atg2b Gskip^+/−^* and *Atg2b Gskip^−/−^* at E13.5. Nuclear fragmentation is detected in the mutant liver (arrows in inset). Bar, 20 μm. Cleaved caspase-3-positive cells were counted in the indicated genotype liver sections at E11.5 to E14.5 and are plotted in the graph. Horizontal bars indicate mean values. (E) Percentages (left) and total numbers (right) of living cells in livers of fetal mice at E13.5. (F) Percentage of Ter119^+^ erythroid cells among living cells. Numbers of embryos used for each analysis are shown in parentheses. *, *P < *0.05, determined by Mann-Whitney *U* test. ns, not significant.

All *Atg2b Gskip^−/−^* mice from *Atg2b Gskip^+/−^* intercrosses died *in utero*, while *Atg2b Gskip* heterozygous (*Atg2b Gskip^+/−^*) mice were born healthy and fertile, with no noticeable pathological phenotype for at least 2 years. Analysis of *Atg2b Gskip^−/−^* embryos at different developmental stages showed that the majority of embryos died before embryonic day (E) 16.5 (Table 1). The *Atg2b Gskip^−/−^* embryos were not significantly smaller than their heterozygous littermates, but exhibited a severe anemic phenotype, with pale skin and subcutaneous edema along the back. Also, the mutant embryos showed exencephaly (Fig. 3A and B). Meanwhile, mice with single knockout of *Atg2b* were born at the expectedendelian frequency (Table 1) and were viable and fertile. Like other knockout mice for *Atg* genes that have homologues (29), these knockout mice showed no noticeable pathological phenotypes for 1 year. *Gskip* knockout mice were also born at the predicted Mendelian frequency (Table 1) and were viable and fertile, which is inconsistent with the study by Deak et al. showing neonatal death (27). This discrepancy may be due to a difference in the targeting region used. Deak’s group designed the targeting vector to delete approximate 1,500 bp, including exon2 and introns 1 and 2 (27). The targeting was accompanied by a 1,500-bp deletion about 15 kbp upstream of exon1 of *Atg2b* (Fig. 1), and it may have decreased and/or abolished *Atg2b* gene expression, as with targeting exon1 of *Gskip*. These results indicated that knocking out both *Atg2b* and *Gskip*, rather than just one of them, causes serious developmental defects in mice.

**TABLE 1:** Genotype analyses of *Atg2b Gskip^+/^*^−^, *Atg2b^+/^*^−^ and *Gskip^+/^*^−^ intercross progeny

DNA source	No. of progeny with genotype:								
	*Atg2b Gskip* ^+/+^	*Atg2b Gskip* ^+/−^	*Atg2b Gskip* ^−/−^	*Atg2b* ^+/+^	*Atg2b* ^+/−^	*Atg2b* ^−/−^	*Gskip* ^+/+^	*Gskip* ^+/−^	*Gskip* ^−/−^
Amnion (E11.5)	1	10	4						
Amnion (E12.5)	5	8	4						
Amnion (E13.5)	8	19	8						
Amnion (E14.5)	7	13	6						
Amnion (E15.5)	1	7	6						
Amnion (E16.5)	5	8	1						
Tail (P0)	25	39	0						
Tail (4 weeks old)				26	44	23	20	29	15

### Phenotype of *Atg2b Gskip* double-knockout embryos.

We focused on hematological studies in the mutant mice since hematopoietic progenitor differentiation is promoted in patients with germ line duplication of *ATG2B* and *GSKIP* (11). Liver size in *Atg2b Gskip^−/−^* mice was smaller than that in control mice (Fig. 3B). Histological analysis using Meyer’s hematoxylin and eosin (H&E) staining showed nuclear fragmentation of cells in the livers of double-knockout embryos, but not in the livers of embryos with single knockout of either *Atg2b* or *Gskip* (Fig. 3C). To examine whether the loss of both *Atg2b* and *Gskip* caused cell death, we carried out immunohistochemical analysis using an antibody against cleaved caspase-3, a hallmark of apoptosis. A marked increase in the number of cleaved caspase-3-positive cells was noted in the livers of *Atg2b Gskip^−/−^* embryos compared to those of control embryos (Fig. 3D). Furthermore, the viable cells in E13.5 fetal livers were significantly decreased in *Atg2b Gskip^−/−^* embryos compared to those in *Atg2b Gskip^+/+^* and *Atg2b Gskip^+/−^* embryos, and consequently the absolute number of living cells in *Atg2b Gskip^−/−^* embryos was decreased (Fig. 3E). We also found that the frequency of Ter119-positive erythroid cells in the living cells was significantly reduced in *Atg2b Gskip^−/−^* embryos compared to those in *Atg2b Gskip^+/+^* and *Atg2b Gskip^+/−^* embryos (Fig. 3F). Since erythropoiesis is a matter of the highest priority in fetal hematopoiesis, and presumably in fetal development, we consider that the disturbed erythropoiesis might cause fetal liver hypoplasia and life-threatening anemia in *Atg2b Gskip*^−/−^ embryos.

### Concomitant reduction of *Atg2b* and *Gskip* genes causes a decrease of hematopoietic stem cells.

**FIG 4:**
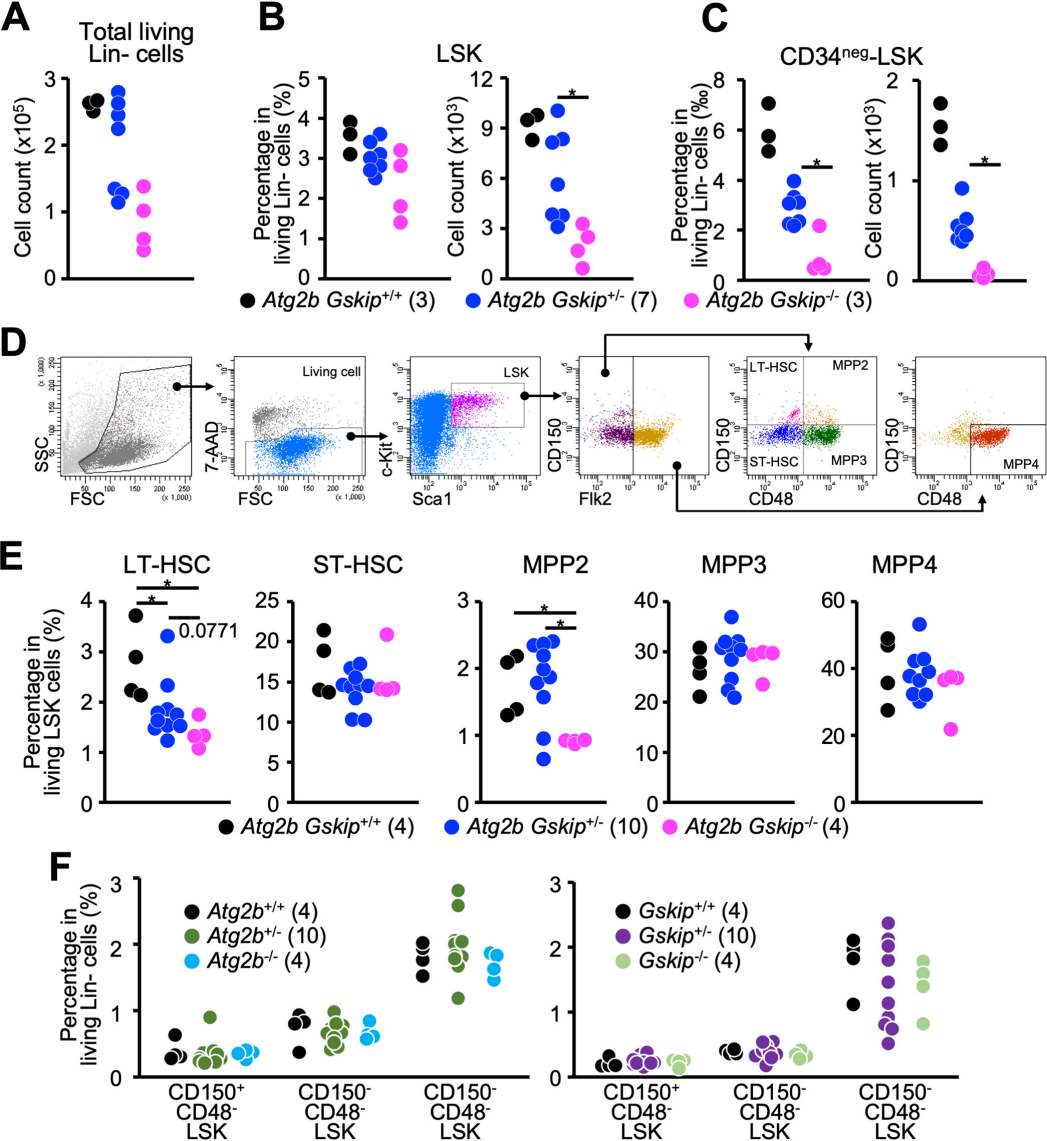
Impairment of hematopoiesis in E13.5 *Atg2b Gskip^−/−^* embryos. (A) Absolute number of living Lin*^−^* cells per liver. (B) Percentage of LSK cells in living Lin*^−^* cells (left) and absolute number of LSK cells per liver (right). (C) Percentage of CD34^neg^-LSK cells in living Lin*^−^* cells (left) and absolute number of CD34^neg^-LSK cells per liver (right). (D) Representative gating strategy used to identify LT-HSC, ST-HSC, MPP2, MPP3, and MPP4. (E) Percentages of LT-HSCs, ST-HSCs, MPP2, MPP3, and MPP4 cells in the LSK fraction. (F) Percentages of CD150^+^ CD48*^−^* LSK, CD150*^−^* CD48*^−^* LSK, and CD150^+^ CD48^+^ LSK cells among living Lin*^−^* cells in E13.5 *Atg2b^−/−^* (left) and *Gskip^−/−^* embryos (right). Numbers of embryos used for each analysis are shown in parentheses. *, *P < *0.05, determined by Mann-Whitney *U* test.

The number of living lineage-negative (Lin^−^) cells in *Atg2b Gskip^−/−^* embryonic livers decreased compared to that in wild-type embryos (Fig. 4A). Accordingly, the number of living Lin^−^ Sca1^+^ c-Kit^+^ (LSK) cells was reduced in *Atg2b Gskip^−/−^* embryos, although no significant differences in the frequency of LSK cells in Lin^−^ cells were observed among the three genotype groups (Fig. 4B). On the other hand, Lin^−^ CD34^−^ Sca1^+^ c-Kit^+^ (CD34^neg^-LSK) cells, which have the potential for long-term lymphohematopoietic reconstitution activity (30), were markedly reduced not only in number but also in frequency in the living Lin^−^ cells in *Atg2b Gskip^−/−^* embryos compared to those in the wild type (Fig. 4C). It is worth noting that the numbers of living Lin^−^ cells and living LSK cells differed between *Atg2b Gskip^+/−^* embryos; some of these embryos had comparable numbers of cells to those of wild-type embryos, whereas the numbers in other embryos were decreased to the level seen in *Atg2b Gskip^−/−^* embryos (Fig. 4A and B, right). Furthermore, the absolute number and frequency of CD34^neg^-LSK cells were decreased in *Atg2b Gskip^+/^*^−^ embryos compared to those in wild-type embryos (Fig. 4C).

To further clarify the hematopoietic phenotype caused by concomitant reduction of *Atg2b* and *Gskip* genes, we prepared 18 embryos at E13.5 by *in vitro* fertilization of male and female *Atg2b Gskip^+/^*^−^ mice and performed flow cytometric analysis. We divided LSK cells into five fractions, specifically long-term HSCs (LT-HSCs), short-term HSCs (ST-HSCs), and three distinct subpopulations of multipotent progenitor cells (MPP2, MPP3, and MPP4), based on the expression profiles of CD150, CD48, and CD135 (31) (Fig. 4D). MPP2, MPP3, and MPP4 cells are defined as MPPs which are developmentally biased toward erythrocyte/megakaryocyte, monocyte/granulocyte, and lymphocyte lineages, respectively (32). As shown in Fig. 4E, the frequency of LT-HSCs within LSK cells significantly decreased in *Atg2b Gskip^+/^*^−^ embryos compared to that in wild-type embryos and was further reduced in *Atg2b Gskip^−/−^* mice (Fig. 4E, left), which was in good agreement with the result shown in Fig. 4C. Thus, HSCs in heterozygous mice appeared to exhibit a haploinsufficiency phenotype. We also found that the frequency of MPP2 cells was significantly reduced in *Atg2b Gskip^−/−^* mice but was maintained in *Atg2b Gskip^+/−^* embryos. There were minimal differences in the frequencies of ST-HSCs, MPP3, and MPP4 among the three genotypes. We confirmed that there were no substantial differences in the numbers of HSC subpopulations in embryos with single knockout of either *Atg2b* or *Gskip* (Fig. 4F).

**FIG 5:**
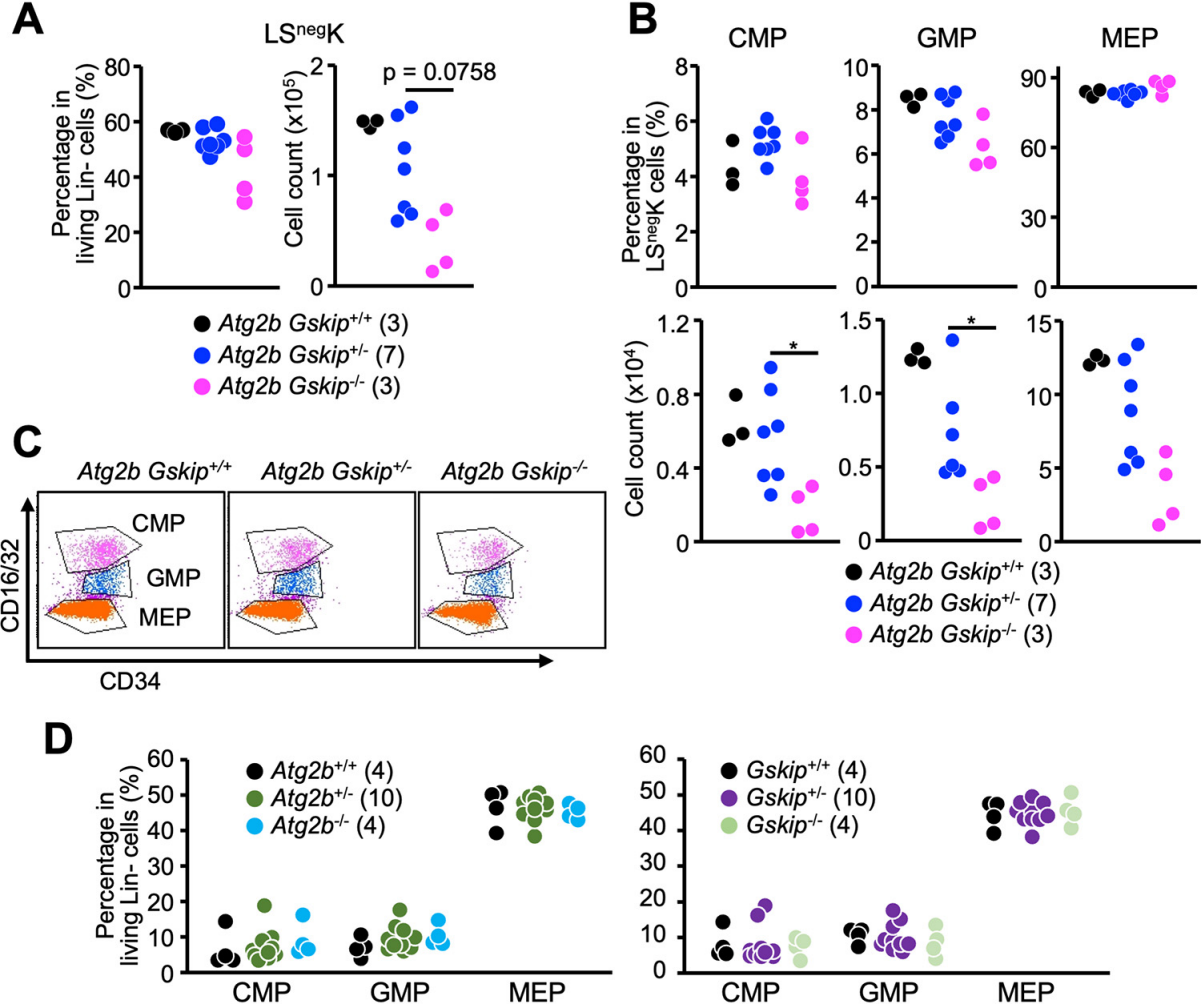
Flow cytometric analysis of CMPs, GMPs, and MEPs in embryos. (A) Percentage of LS^neg^ cells among living Lin*^−^* cells (left) and absolute number of LS^neg^ cells per liver (right). (B) Percentages of CMPs, GMPs, and MEPs in LS^neg^K cells (upper subpanels) and absolute numbers of CMP, GMP, and MEP cells per liver (lower subpanels). (C) Representative dot plots of LS^neg^ cells showing CD16/32 and CD34 expression. CMPs, GMPs, and MEPs are defined as indicated. (D) Percentages of CMP, GMP, and MEP cells among living Lin*^−^* cells in E13.5 *Atg2b^−/−^* (left) and *Gskip^−/−^* embryos (right). Numbers of embryos used for each analysis are shown in parentheses. *, *P < *0.05, determined by Mann-Whitney *U* test.

We next analyzed Lin*^−^* Sca1*^−^* c-Kit^+^ hematopoietic precursor (LS^neg^K) cells and found that the relative percentage of these cells among living Lin*^−^* cells were largely similar among the genotypes (Fig. 5A), suggesting that the remaining HSCs in *Atg2b Gskip^−/−^* fetal livers have the ability to differentiate into hematopoietic progenitor cells. The frequencies of granulocyte-monocyte progenitor (GMP) cells, megakaryocyte-erythroid progenitor (MEP) cells, and common myeloid progenitor (CMP) cells gated on Lin*^−^* cells of *Atg2b Gskip^−/−^* embryos were comparable to those of *Atg2b Gskip^+/−^* and wild-type embryos, although the absolute numbers of hematopoietic precursor cells were reduced in *Atg2b Gskip^−/−^* embryos (Fig. 5B and C). Predictably, embryos of mice deficient in *Atg2b* or *Gskip* alone did not show any abnormalities in hematopoiesis (Fig. 5D). In summary, the concomitant reduction of *Atg2b* and *Gskip* genes led to a haploinsufficiency phenotype while complete loss of either the *Atg2b* or *Gskip* gene had little impact on the hematopoietic system in mice.

### Hematopoietic analysis of adult mice heterozygous for the *Atg2b Gskip* knockout allele.

**FIG 6:**
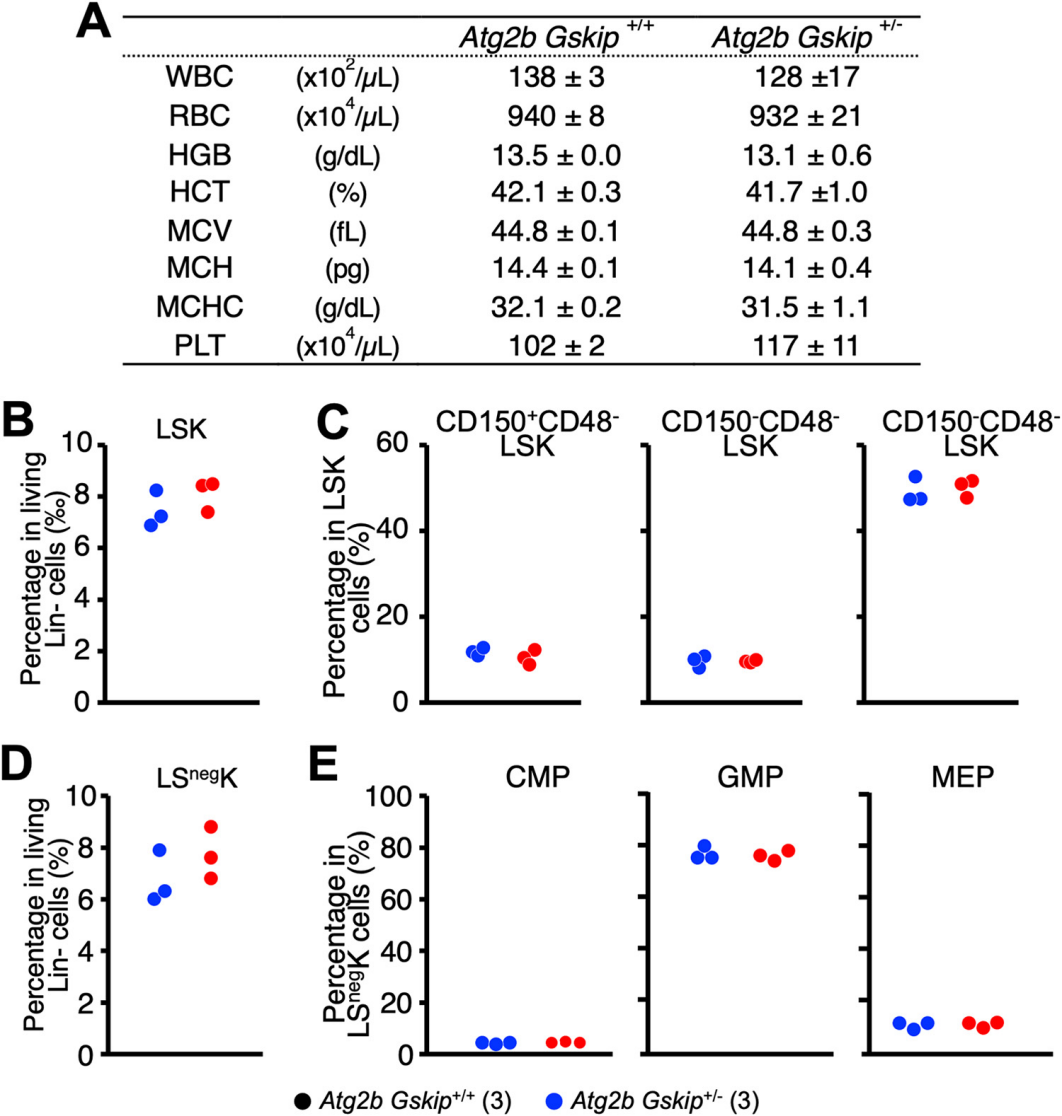
Normal hematopoiesis in heterozygous *Atg2b Gskip*^+/^*^−^* adult mice. (A) Hematopoietic indices of three *Atg2b Gskip*^+/+^ and three *Atg2b Gskip*^+/^*^−^* mice at 10 weeks old. Mice were bled from the retro-orbital plexus under anesthesia. (B to E) Summary of flow cytometric analyses of three *Atg2b Gskip*^+/+^ and three *Atg2b Gskip*^+/^*^−^* mice at 10 weeks old. Percentage of CD34^neg^-LSK cells among Lin*^−^* cells (B), the percentages of LT-HSCs, ST-HSCs, and MPPs among CD34^neg^ LSK cells (C), percentage of LS^neg^ cells among Lin*^−^* cells (D), and percentages of CMPs, GMPs, and MEPs in LS^neg^ cells (E) are shown. Numbers of embryos used for each analysis are shown in parentheses.

Despite the finding that the absolute numbers of LSK, LS^neg^K, CMP, GMP, and MEP cells differed between *Atg2b Gskip^+/−^* heterozygous mice (Fig. 4B and Fig. 5A and B), these mice were born at the expected Mendelian frequency. Furthermore, the hematopoietic indices of adult *Atg2b Gskip^+/−^* mice had the same values as those of *Atg2b Gskip^+/+^* mice (Fig. 6A).

We next performed flow cytometric analyses of the bone marrow (BM) of adult *Atg2b Gskip^+/−^* mice. In contrast to the findings of embryonic hematopoiesis, the frequencies of HSC/progenitor subpopulations in individual *Atg2b Gskip^+/−^* mice were virtually equivalent to those in wild-type mice (Fig. 6B to E). While fetal HSCs have the capacity to rapidly self-renew and produce progeny in order to support hematopoiesis in developing embryos, most adult HSCs are in quiescence (33, 34). We speculate that the differences between embryonic and adult HSCs are partly due to differences in the nature of these HSCs.

### Profiling of *ATG2B*- and *GSKIP*-deficient cells.

**FIG 7:**
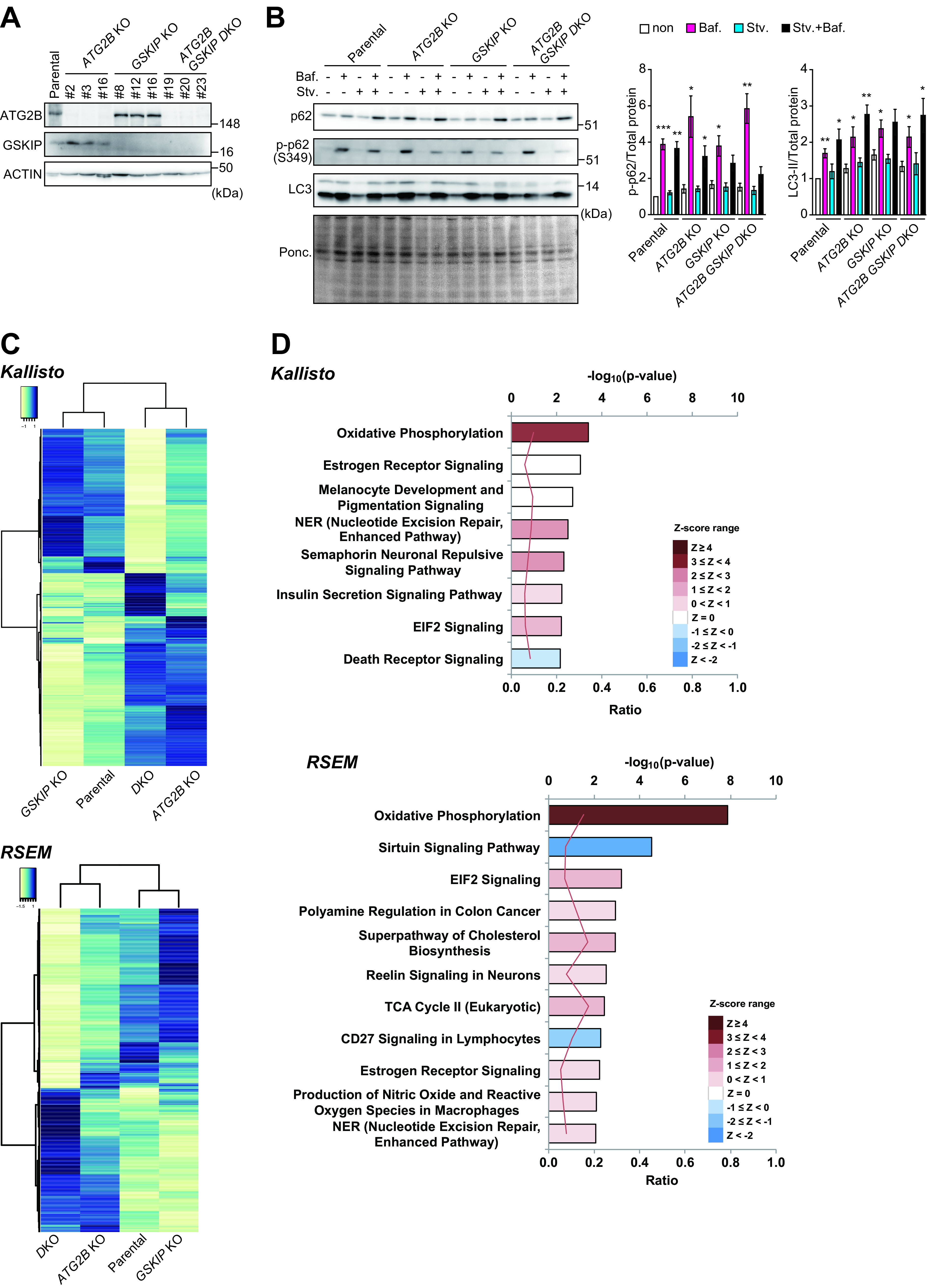
Dissection of autophagic activity and gene expression in *ATG2B*–*GSKIP*-deficient K562 cells. (A and B) Immunoblot analysis. Parental, *ATG2B*-, *GSKIP*-, and *ATG2B*–*GSKIP*-deficient K562 cells were lysed, and then cell lysates were subjected to SDS-PAGE, followed by immunoblotting with the indicated antibodies (A). Parental, *ATG2B*-, *GSKIP*-, and *ATG2B*–*GSKIP*-deficient K562 cells were cultured under nutrient-rich (−) or nutrient-deprived (stv.) conditions in the presence or absence of bafilomycin A1 (Baf A_1_). Cell lysates were subjected to SDS-PAGE, followed by immunoblot analysis with the indicated antibodies and Ponceau staining (B). Graphs show the levels of LC3-II and S349-phosphorylated p62 per total protein, estimated by Ponceau staining. Data shown are representative of four separate experiments. (C) Gene clustering. RNA-seq analysis was performed using wild-type (*n *= 3), *ATG2B* single-knockout (*n *= 3), *GSKIP* single-knockout (*n *= 3), and *ATG2B GSKIP* double-knockout K562 cells (*n *= 3). Hierarchical clustering was performed on differentially expressed genes and is presented as a heatmap. Right and left heatmaps were created using Kallisto and RSEM, respectively. (D) Ingenuity pathway analysis of the RNA-seq data was used to predict signaling pathway activity. The pathways for which the *P* value was ≤0.01 and the Z-score could be calculated are shown. The upper axis shows −log_10_(*P* value), and the lower axis shows the ratio between the number of differentially expressed genes and the number of genes in each pathway. The Z-score, which predicts activation (positive values) or inhibition (negative values) of canonical pathways, is shown by the color scale. An absolute Z-score of ≥2 is considered significant.

It has been reported that autophagy is critical for proper hematopoiesis (35, 36), HSC mobilization (37), and the survival of adult HSCs in the setting of acute metabolic stress (38). Autophagy also preserves the regenerative capacity of HSCs through metabolic suppression (39). Thus, it is plausible that loss of *ATG2B* and *GSKIP* is associated with decreased autophagy, resulting in a deficit in the blood development system. We examined whether the loss of *ATG2B*, *GSKIP*, or both had an effect on autophagy. To this end, we used CAS9-CRISPR technology to delete one or both genes in the human leukemia cell line K562 (Fig. 7A). The levels of LC3-II and S349-phosphorylated p62, both of which are degraded by autophagy, were comparable between both mutant K562 cell types and wild-type K562 cells (Fig. 7B). An autophagy flux assay with bafilomycin A_1_ (Baf A_1_), which is an inhibitor of lysosomal acidification, revealed that in both mutant K562 cell types, treatment with Baf A_1_ caused upregulation of LC3-II and S349-phosphorylated p62 to a similar extent to that in parental K562 cells (Fig. 7B), indicating intact autophagy.

Finally, we conducted transcriptome sequencing (RNA-seq) analysis with wild-type, *ATG2B*, *GSKIP*, and *ATG2B GSKIP* double-knockout K562 cells. We used two pipelines, Kallisto and RNA-Seq by Expectation Maximization (RSEM), to quantify 35,619 transcripts and 26,670 genes, respectively. Multiple comparisons after analysis of variance (ANOVA) revealed that the numbers of genes differentially expressed in the double-knockout K562 cells were 731 with Kallisto and 730 with RSEM. Hierarchical clustering exhibited gene clusters uniquely expressed in each mutant K562 cell type (Fig. 7C). Ingenuity pathway analysis (IPA) with transcripts and genes specified by Kallisto and RSEM identified a significant increase in oxidative phosphorylation activity in *ATG2B GSKIP* double-knockout K562 cells (Fig. 7D; see also Table S1 in the supplemental material). Taken together, we concluded that while loss of *ATG2B* and *GSKIP* had a minimal effect on autophagy, it was accompanied by increased gene expression of enzymes involved in oxidative phosphorylation.

**FIG 8:**
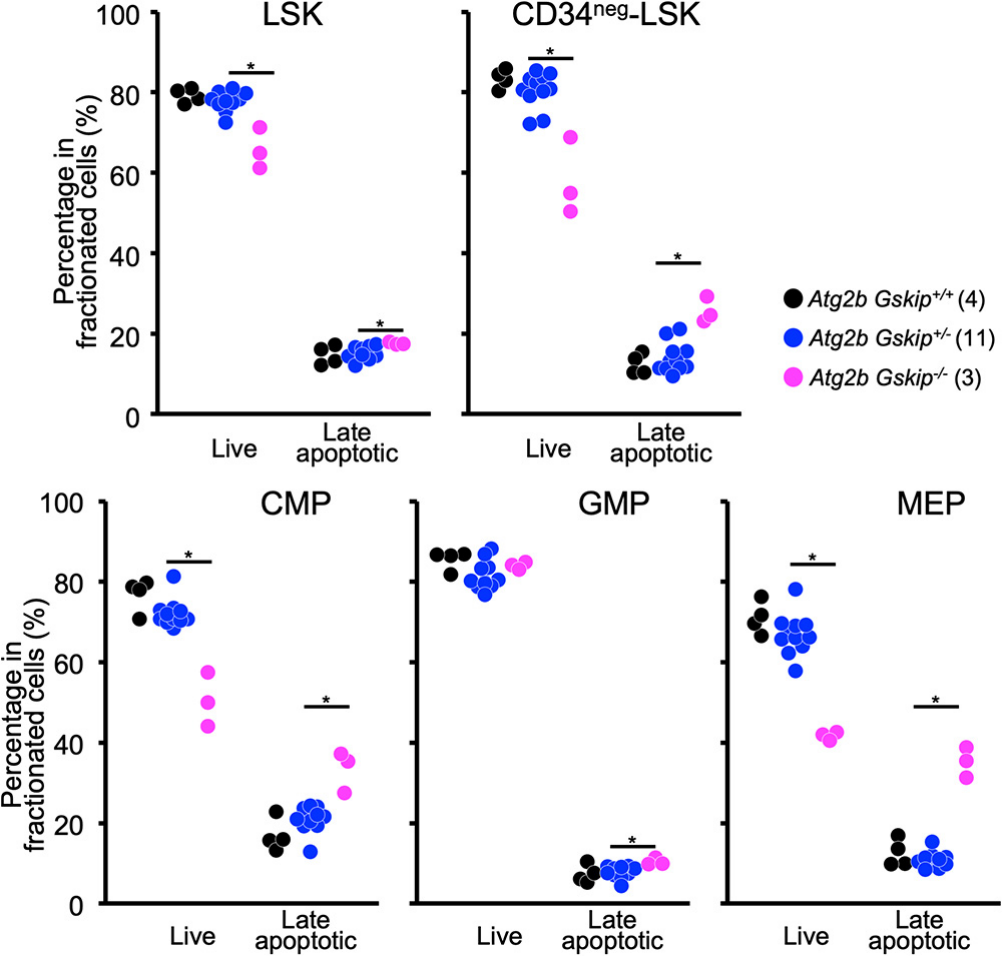
Frequencies of living and late apoptotic cells. Frequencies of living and late apoptotic cells gated in LSK, CD34^neg^-LSK, CMP, GMP, and MEP fractions are presented. Living and late apoptotic cells were defined as annexin V^−^ 7-AAD^−^ and annexin V^+^ 7-AAD^+^, respectively. Four *Atg2b Gskip^+/+^*, 11 *Atg2b Gskip^+/^*^−^, and three *Atg2b Gskip^−/−^* embryos were used. Numbers of embryos used for each analysis are shown in parenthesis. *, *P < *0.05, determined by Mann-Whitney *U* test.

Excessive oxidative phosphorylation may lead to an increase in reactive oxygen species (ROS) and finally cause cell death by apoptosis (40). We therefore evaluated the levels of apoptosis in hematopoietic stem/progenitor cells. The concurrent loss of *Atg2b* and *Gskip* increased the number of annexin V^+^ 7-aminoactinomycin D (7-AAD)^+^ late apoptotic cells in LSK, CD34^neg^-LSK, CMP, GMP, and MEP fractions (Fig. 8). In addition, the numbers of annexin V^−^ 7-AAD^−^ living cells in these lineages were markedly decreased in *Atg2b Gskip^−/−^* mice compared to those in *Atg2b Gskip^+/+^* mice, except in the GMP fraction (Fig. 8), suggesting that the apoptotic cell death of HSCs and immature progenitors might be at least in part relevant to the decreased number of hematopoietic cells in *Atg2b Gskip^−/−^* mice. In contrast to these findings, neither an increase in apoptotic cells nor a decrease in live cells was evident in these lineages in the fetal livers of *Atg2b Gskip^+/^*^−^ mice (Fig. 8), although the number of HSCs was significantly decreased in *Atg2b Gskip^+/^*^−^ mice (Fig. 4). We consider that concomitant haploinsufficiency of *Atg2b* and *Gskip* may contribute to maintaining the pool size of HSCs.

## DISCUSSION

It has been suggested that regions with relatively hypoxic conditions harbor the most quiescent HSCs, rather than other hematopoietic progenitors and lineage-committed precursor cells (41–43). Glycolysis is the most important metabolic pathway in quiescent HSCs, while increases in reactive oxygen species (ROS) levels due to switching of the energy generation mode to mitochondrial respiration induces stem cell differentiation (44–46). The metabolic status in HSCs is determined by many signaling pathways (44, 47). Therefore, the regulation of metabolic conflict between glycolysis and mitochondrial respiration is a key to balancing quiescence and proliferation/differentiation in HSCs. We showed here that the concomitant reduction of *Gskip* and *Atg2b* led to reduced pool size of HSCs in mice, while there were no remarkable changes in the hematopoietic system caused by single loss of either *Gskip* or *Atg2b*. Simultaneous knockout of *Gskip* and *Atg2b* increased the gene expression of enzymes involved in oxidative phosphorylation. We consider that the energy metabolism pathway of HSCs may be shifted from glycolysis to oxidative phosphorylation in mitochondria by the concomitant deficiency of Gskip or Atg2b functions, thus altering the fate of HSCs.

In this regard, GSKIP is an anchoring protein for GSK3β and PKA, which are involved in the regulation of multiple cellular signaling pathways, including the Wnt/β-catenin and PI3K/AKT/mTOR pathways (23–26). Accumulating evidence suggests that the Wnt/β-catenin and mTOR pathways play an important role in determining the fate of normal HSCs, including quiescence, self-renewal, and differentiation statuses (48, 49), and also help determine the nature of MPN stem cells (3–6). We speculate that although mice with loss of *Gskip* alone showed no particular phenotype in the hematopoietic system, probably due to functional redundancy in the signaling network in mice, deficiency of *Gskip* with concomitant ablation of *Atg2b* may be over the redundancy threshold. Since autophagy is required for erythroid differentiation (35, 36), as well as for the maintenance and mobilization of HSCs (37, 38), we considered that loss of *ATG2B* suppresses autophagic activity, although not completely, thus exerting a synergistic effect with *Gskip* ablation on the pool size of HSCs. However, autophagic activity in *ATG2B* knockout and *ATG2B GSKIP* knockout K562 cells was comparable to that in parental K562 cells (Fig. 7B), probably due to redundant function of ATG2A. Actually, we verified the expression of ATG2A protein in *ATG2B* knockout and *ATG2B GSKIP* knockout K562 cells and also demonstrated similar copy numbers of both *Atg2a* and *Atg2b* transcripts in mouse embryonic LSK cells (data not shown). ATG2A is localized to isolation membranes/phagophores but also to lipid droplets, and deficiency of both *ATG2A* and *ATG2B* causes abnormal enlargement of these droplets (14, 50). Since lipid droplets have the ability to scavenge ROS (51–53), cells lacking ATG2B and GSKIP might become vulnerable to ROS.

We identified the haploinsufficiency phenotype in *Atg2b Gskip^+/^*^−^ fetal livers, although we could not observe differences in BM hematopoiesis between *Atg2b Gskip^+/^*^−^ and *Atg2b Gskip^+/+^* mice. This might be due to the differences between fetal and adult HSCs—self-renewal and proliferation are accelerated in fetal HSCs to produce abundant hematopoietic progeny in the developing embryos, while adult HSCs are largely quiescent and only occasionally enter the cell cycle to maintain hematopoietic homeostasis (33, 34). Previous work demonstrated that oxidative metabolic pathways were activated in fetal HSCs compared with adult HSCs, and consequently, total ROS levels were significantly higher in fetal livers than in BM (54). We speculate that ROS levels in HSCs in *Atg2b Gskip^+/^*^−^ embryos are prone to reach the threshold that enables HSCs to change their cell fate, leading to the phenotypic heterogeneity observed in individual *Atg2b Gskip^+/^*^−^ embryos. In contrast, baseline ROS levels in adult HSCs are predicted to be substantially increased in *Atg2b Gskip^+/^*^−^ HSCs, with ROS having limited influence. Although we could not identify the haploinsufficiency phenotype in A*tg2b Gskip^+/^*^−^ adult mice at 10 weeks of age, we speculate that increased baseline ROS levels may facilitate stem cell ageing, accompanied by the characteristic features of less frequent quiescence, myeloid bias, and DNA damage accumulation in HSCs (55).

Our experiments indicated a genetic interaction between *Atg2b* and *Gskip* and a synergistic effect of ATG2B and GSKIP on the maintenance of both HSCs and hematopoietic progenitor cells by a mechanism that is currently unknown and does not involve autophagy regulation. Our observations may provide insight into the molecular mechanisms of familial MPN and AML.

## MATERIALS AND METHODS

### Cell culture.

MEFs were grown in Dulbecco’s modified Eagle medium (DMEM) containing 10% fetal bovine serum (FBS), 5 U/mL penicillin, and 50 μg/mL streptomycin. K562 cells (JCRB0019) were grown in RPMI 1640 medium containing 10% fetal bovine serum, 5 U/mL penicillin, and 50 μg/mL streptomycin. To generate *ATG2B* and *GSKIP* knockout K562 cells, each guide RNA designed using the CRISPR design tool (http://crispr.mit.edu/) was subcloned into pX330-U6-Chimeric_BB-CBh-hSpCas9 (catalog no. 42230; Addgene; deposited by Feng Zhang’s lab), a human codon-optimized SpCas9 and chimeric guide RNA expression plasmid. K562 cells were cotransfected with vectors pX330 and pEGFP-C1 (catalog no. 6084-1; TaKaRa Bio, Inc., Shiga, Japan), and cultured for 2 days. Thereafter, green fluorescent protein (GFP)-positive cells were sorted and expanded. Loss of *ATG2B* and *GSKIP* was confirmed by heteroduplex mobility assays followed by immunoblot analysis with anti-ATG2B and anti-GSKIP antibodies.

### Generation of genetically modified mice.

The *Atg2b*-*Gskip*-targeting vector was constructed by insertion of *loxP* sequences prior to and after exon1 of the *Atg2b* gene (prior to and inside exon1 of the *Gskip* gene). The targeting vectors were electroporated into mouse RENKA ES cells, selected with G418 (250 μg/mL; Invitrogen, San Diego, CA), and then screened for homologous recombinants by Southern blotting. Southern blot analysis in *Atg2b Gskip^flox^* mice was performed by digesting genomic DNA with SpeI (TaKaRa Bio, Inc.) or EcoRI (TaKaRa Bio, Inc.), followed by hybridization to detect wild-type 16.5-kb and flox 9-kb bands or wild-type 10.5-kb and flox 8.9-kb bands.

To generate single *Atg2b*- and *Gskip*-deficient mice by CRISPR-Cas technology, CRISPR RNAs (crRNAs) were designed to recognize target sites (exon40 of *Atg2b*, 5′-AAGGATGGCCGTATCGTCAG-3′; exon2 of *Gskip*, 5′-GGAAACAGACTATAATCCCG-3′). The synthetic crRNAs (Alt-R CRISPR-Cas9 crRNA), *trans*-activating CRISPR RNA (tracrRNA) (Alt-R CRISPR-Cas9 tracrRNA), and Cas9 protein (Alt-R S.p. Cas9 Nuclease 3NLS) were purchased from Integrated DNA Technologies, Inc. (IDT; Coralville, IA). The CRISPR/Cas9 solution was prepared as previously described (56), with minor modifications. Briefly, lyophilized crRNAs and tracrRNA were resuspended in nuclease-free duplex buffer (IDT) to a concentration of 240 μM. Equal volumes of crRNA and tracrRNA were combined, heated at 95°C for 5 min, and then placed at room temperature (RT) for about 10 min to allow formation of crRNA-tracrRNA duplex. The crRNA-tracrRNA duplex was mixed with Cas9 protein to form a ribonucleoprotein complex in Opti-MEM (Thermo Fisher Scientific, Waltham, MA). The final concentrations of Cas9 protein and crRNA-tracrRNA duplex were 1 μg/μL and 30 μM, respectively. To induce CRISPR/Cas9-mediated deletion/insertion, we applied a recently developed method called improved genome editing via oviductal nucleic acid delivery (i-GONAD) (56). Approximately 1.5 μL of CRISPR/Cas9 solution was injected into the oviductal lumens of female C57BL/6N mice at day 0.7 of pregnancy. Immediately after the injection, the oviduct regions were grasped with a tweezer-type electrode (catalog no. CUY652-3; Nepa Gene Co., Ltd., Chiba, Japan) and then electroporated using the NEPA21 square-wave pulse generator (Nepa Gene). The electroporation parameters used were as previously described (56). Pregnant female mice were allowed to deliver their pups. Tail biopsies of pups were performed for genomic DNA isolation.

### RT-qPCR.

Using the Transcriptor first-strand cDNA synthesis kit (Roche Applied Science, Indianapolis, IN), cDNA was synthesized from 1 μg of total RNA. Real-time quantitative reverse transcription-PCR (RT-qPCR) was performed using the LightCycler 480 probes master mix (Roche Applied Science) on a LightCycler 480 instrument (Roche Applied Science). Signals from mouse samples were normalized against *Gusb* (β-glucuronidase) mRNA. The sequences of the primers used were as follows: *Atg2b* Left, TTTTGCACCAAAAACAGTCG; *Atg2b* Right, CGTCGTCAACCATGTCTTTATC; *Gskip* Left, GGAGCAAAGGAAAGGAACAGA; and *Gskip* Right, TCAAAACATAGCCCACCA.

### Immunoblot analysis.

Mouse livers and brains were homogenized in 0.25 M sucrose, 10 mM 2-(4-[2-hydroxyethyl]-1-piperazinyl)ethanesulfonic acid (HEPES) (pH 7.4), and 1 mM dithiothreitol (DTT). Cells were lysed with TNE buffer (20 mM Tris-HCl [pH 7.5], 0.5% Nonidet P-40, 150 mM NaCl, 1 mM ethylenediaminetetraacetic acid [EDTA]) containing 1 mM DTT and protease inhibitor cocktail (Roche Applied Science). Samples were subjected to SDS-PAGE and then transferred to a polyvinylidene difluoride membrane (IPVH00010; Merck). Antibodies against Atg2b (catalog no. 25155-1-AP, 1:500; Proteintech Group, Inc., Rosemont, IL), Gskip (catalog no. PA5-60218, 1:500; Thermo Fisher Scientific), LC3B (catalog no. 2775, 1:500; Cell Signaling Technology, Boston, MA), and actin (MAB1501R, 1:1,000; Merck Millipore, Burlington, MA) were purchased from the indicated suppliers. Blots were incubated with horseradish peroxidase-conjugated goat anti-mouse IgG (H+L) (catalog no. 115-035-166; Jackson ImmunoResearch Laboratories, Inc., West Grove, PA), goat anti-rabbit IgG (H+L) (catalog no. 111-035-144; Jackson ImmunoResearch Laboratories, Inc.), or goat anti-guinea pig IgG (H+L) antibody (catalog no. 106-035-003; Jackson ImmunoResearch Laboratories, Inc.), and visualized by chemiluminescence. Band density was measured using Multi Gauge v3.2 software (Fujifilm Corporation, Tokyo, Japan).

### Histological analysis.

Excised liver tissues were fixed by immersion in 0.1 M phosphate buffer (PB; pH 7.4) containing 4% paraformaldehyde and 4% sucrose. Embryos were delivered, and their livers were fixed by immersion in 0.1 M PB (pH 7.4) containing 4% paraformaldehyde and 4% sucrose. Each liver was carefully dissected and processed for paraffin embedding, and then sections were prepared for hematoxylin and eosin staining. Images were captured with a BX51 microscope (Olympus, Tokyo, Japan). For detection of apoptotic cells, sagittal serial sections of 3-μm thickness were prepared for each embryo. Three to six sections were selected at intervals of 90 to 160 μm and immunostained with anti-cleaved caspase-3 (catalog no. 9661, 1:500; Cell Signaling Technology). Images were captured with a BX53 microscope (Olympus). For quantification, the number of cleaved caspase-3-positive cells per square millimeter in each liver section was counted.

### Flow cytometry.

For cell population analyses, lineage depletion was performed using a cocktail of biotinylated antibodies against Ter119, B220, Gr1, CD4, CD8, and CD127, followed by their removal using Dynabeads M-280 streptavidin-conjugated magnetic beads (Thermo Fisher Scientific). Lin^−^ hematopoietic stem and progenitor cells (HSPCs) were stained with a combination of allophycocyanin (APC)-conjugated anti-CD34, fluorescein isothiocyanate (FITC)-conjugated anti-CD48, phycoerythrin (PE)-conjugated or brilliant violet 510 (BV510)-conjugated anti-CD150, PE-conjugated anti-CD135, APC-eFluor 780 (APC-eF780)-conjugated anti-c-Kit, PE-cyanin 7 (PE-Cy7)-conjugated anti-CD16/32, and brilliant violet 421 (BV421)-conjugated anti-Sca1. Dead cells were excluded by 7-AAD. For apoptotic cell analysis, lineage-negative HSPCs were stained with a combination of APC-conjugated anti-CD34, PE–Cy7-conjugated anti-CD16/32, APC–eF780-conjugated anti-c-Kit, BV421-conjugated anti-Sca1, FITC-conjugated anti-annexin V, and 7-AAD. Stained cells were analyzed with a BD FACSAria II flow cytometer and BD FACSDiva software (Becton, Dickinson, Franklin Lakes, NJ). Information regarding antibodies is available upon request.

### Differential expression analysis.

Read mapping was conducted according to the GRCh38 reference genome using Spliced Transcripts Alignment to a Reference (STAR) software v2.7.7a (57), and then gene expression and isoform expression were quantified by RSEM v1.3.3 (58). RNA-seq analysis was also performed by Kallisto using Ensembl Homo sapiens GRCh38 cDNA transcripts (release 104) for indexing. Transcript-level quantification was examined by Kallisto version v0.46.2 (59) using 100 bootstrap samples. Excepted counts of 12 samples in four groups on the gene level, obtained with RSEM or Kallisto, were used for differential expression analysis with DESeq2 (60). Estimated counts of transcript level obtained with Kallisto were mapped and switched using Ensembl GRCh38 release 98. In detail, the significance of the change in deviance between the four groups was tested by the likelihood ratio test, and *post hoc* tests between the three knockout groups and the wild-type group were conducted by Wald’s test. All *P* values generated by the above-described tests were adjusted by the Hochberg method (61). Differentially expressed genes present only in double-knockout mice were defined as those that were significant in double-knockout versus wild-type mice and nonsignificant in both of the single-knockout mice versus wild-type mice, with a significance level set to 5%. Transcripts per million (TPM) values of differentially expressed genes occurring only in double-knockout mice were used to explore specially expressed gene clusters for each group by hierarchical clustering, with the Euclidean distance method and Ward clustering method. The clustering results are shown by heatmap. All statistical tests and preparation of graphics were performed with R v4.0.0 software (https://www.r-project.org/). Ingenuity Pathway Analysis (IPA, released September 2021; Qiagen Redwood City, CA) was used to identify significant canonical pathways involving the differentially expressed genes enriched using the above two methods.
